# Singleplex real-time PCR and duplex PCR platform for the rapid detection of hypervirulent *Klebsiella pneumoniae*

**DOI:** 10.3389/fvets.2025.1611750

**Published:** 2025-07-17

**Authors:** Jiaqi Wang, Yishuai Wu, Liying Zhao, Mengran Xing, Yangyang Huang, Shengjie Peng, Chunyan Xu, Hong Yao, Chenglong Li, Xiang-Dang Du

**Affiliations:** ^1^International Joint Research Center of National Animal Immunology, College of Veterinary Medicine, Henan Agricultural University, Zhengzhou, China; ^2^Ministry of Education Key Laboratory for Animal Pathogens and Biosafety, Henan Agricultural University, Zhengzhou, China; ^3^Ministry of Agriculture and Rural Affairs, Key Laboratory of Quality and Safety Control of Poultry Products, Henan Agricultural University, Zhengzhou, China; ^4^College of Veterinary Medicine, Huazhong Agricultural University, Wuhan, China

**Keywords:** singleplex real-time PCR, duplex PCR, biomarkers, melting curve analysis, hypervirulent *Klebsiella pneumoniae*

## Abstract

Hypervirulent *Klebsiella pneumoniae* (HvKP) is a notorious zoonotic pathogen that poses a significant threat to public health, as it can cause severe infections with high morbidity and mortality among young and healthy individuals. Commonly, a positive string test is primarily used to identify HvKP strains, but which is laborious and time-consuming. A rapid assay to identify HvKP is needed for public health personnel to track the spread of these strains and provide timely efforts for their control. Given this context, rapid SYBR Green I-based singleplex real-time PCR assays were developed for defining HvKP on the basis of the biomarkers *iroB*, *iucA*, *peg-344* and plasmid-borne *rmpA*, respectively. These four singleplex PCR assays all displayed a high degree of linearity (*R*^2^ > 0.99) in the range of 10^4^ to 10^9^ cfu/mL, and the limit of detection (LOD) was 10^3^ cfu/mL, which was equivalent to 10 cfu/reaction. To improve the efficiency and reduce the cost of diagnostic testing, SYBR Green I-based duplex PCR melting curve assays were developed with average melting temperatures of 85.0, 87.5, 76.0 and 78.5°C for *iroB*, *iucA*, *peg-344* and plasmid-borne *rmpA*, respectively. The LODs for the developed duplex PCRs for *iroB*/*peg-344*, *iucA*/*peg-344* and *iucA*/*rmpA* combination were 10^4^ cfu/mL (that was 10^2^ cfu/reaction), 10^4^ cfu/mL (that was 10^2^ cfu/reaction) and 10^5^ cfu/mL (that was 10^3^ cfu/reaction), respectively. High specificity was shown when other bacterial pathogens were detected in this study. These assays could be used as rapid, sensitive and specific diagnostic tools for the practical identification of HvKP strains.

## 1 Introduction

*Klebsiella pneumoniae* is an increasingly important opportunistic pathogen in zoonoses that is clinically isolated from a wide range of samples, including the gastrointestinal tract, respiratory tract and urinary tract in both animals, humans and the environment, and commonly causes nosocomial infections, including bacteremia, pneumonia and urinary tract infections (1–4). Unlike classical *K. pneumoniae* (cKP), a distinct pathotype of *K. pneumoniae*, hypervirulent *K. pneumoniae* (HvKP) has emerged as a notorious pathogen capable of causing community-acquired and increasingly healthcare-associated infections, including pyogenic liver abscess and other severe organ and life-threatening infections, among healthy individuals of any age. Alarmingly, carbapenem-resistant HvKP, colistin-resistant HvKP and tigecycline-resistant HvKP have emerged, creating a new challenge in combating these threats, as they are hypervirulent, multidrug-resistant and highly transmissible (5–9). Given the increasing emergence and global dissemination of HvKP, rapidly detecting and continuously surveilling such organism is a priority.

Traditional methods, such as colony morphology, *Galleria mellonella* or mouse lethality assays and serum killing assay, especially the string test, have been widely used for identifying HvKP (10, 11). However, these methods are costly, time-consuming and are not suitable for the rapid detection of HvKP. Moreover, for the widely used string test, studies have shown that not all HvKP isolates are hypermucoviscous, which limits the accuracy of this method to 90% (2, 12). The development of accurate and rapid methods for the identification of HvKP is urgently needed for healthcare providers and public health personnel to track the dissemination of these strains and take timely actions for their control.

With the advantages of accuracy, low cost, rapidity and robustness, PCR-based methods have been widely used for the identification of pathogens (13). Recently, a quintuplex PCR was developed for the detection of HvKP, which targets the plasmid replicon gene, *iroN* (encodes siderophore salmochelin receptor), *iutA* (encodes ferric aerobactin receptor), plasmid-borne *rmpA* and plasmid-borne *rmpA2* (regulates capsular polysaccharide biosynthesis), and agarose gel electrophoresis with GelStar nucleic acid gel stain revealed that the amplicons ranged from 160 bp to 683 bp in size (14). Moreover, a nonuplex PCR was established for the detection of HvKP, which targets genes *allS* (associates with allantoin metabolism), *entB* (involves in siderophore enterobactin biosynthesis), *iutA*, *kfu* (mediates ferric iron uptake), *magA* (relates with the K1 serotype), *mrkD* (encodes type 3 fimbrial adhesin), plasmid-borne *rmpA*, *wzi* (relates with the K2 serotype) and *ybtS* (involves in siderophore yersiniabactin biosynthesis), and agarose gel electrophoresis staining with ethidium bromide revealed that the amplicons were in the range of 242 base pairs (bp) to 1,283 bp (15). However, these traditional PCR methods require agarose gels or other post-PCR processing methods, which always suffering from unsatisfactory limit of detection (LOD), poor specificity and contamination.

As a superior alternative, real-time PCR coupled with melting curve analysis has the advantages of avoiding post-PCR operations, reducing contamination risks, and improving the LOD and specificity has been used for the screening of virulence genes in HvKP, such as the singleplex real-time PCRs which target *iucA* (involves in siderophore aerobactin biosynthesis), *peg-344* (metabolite transporter) and *rmpA* (both plasmid-borne *rmpA*, *rmpA2* and chromosome-borne *rmpA*), respectively (16). However, for the identification of HvKP, in addition to the above-mentioned genes, other genes, such as *iroB* (involves in siderophore salmochelin biosynthesis), *irp2* (involves in siderophore yersiniabactin biosynthesis), *peg-589* (metabolite transport), *peg-1631* (metabolite transport), *terB* (encodes tellurite resistance protein)*, wzx* (K serotype-specific allele) and *wzy* (K serotype-specific allele), are associated with HvKP, which suggests that more real-time PCRs for these related genes should be developed and used simultaneously to differentiate HvKP from cKP (12, 14, 15, 17). Fortunately, studies have proposed that the virulence-related genes *iroB*, *iucA*, *peg-344* and plasmid-borne *rmpA* are biomarkers for the differentiation of HvKP from cKP with diagnostic accuracies greater than 0.96 (12, 18–20). Therefore, in this study, SYBR Green I-based singleplex real-time PCR methods that target biomarkers of HvKP, including *iroB*, *iucA*, *peg-344* and plasmid-borne *rmpA* were developed. Moreover, in terms of cost savings and rapidity, duplex detection methods with melting curve analysis were established. These developed assays with low LODs and high specificities provide efficient tools for identifying and tracking the spread of HvKP in animals, humans and the environment.

## 2 Materials and methods

### 2.1 Samples

HvKP strain 20 K-368 (ST65, K2; *iroB*^+^, *iucA*^+^, *peg-344*^+^, plasmid-borne *rmpA*^+^), and HvKP strain 19 K-29 (*iucA*^+^, plasmid-borne *rmpA*^+^ and *rmpA2*^+^) were isolated and identified as HvKP by the string test (which showed viscous string stretching from the bacterial colony ≥ 5 mm) and sequencing analysis. Other *K. pneumoniae* strains, 19 K-1018, 19 K-1024 and 19 K-1028, were isolated and identified as cKP. Other isolates used in this study, including Gram-negative bacteria, such as *Escherichia coli* EC23030102 and *Salmonella typhimurium* SQ1, and Gram-positive bacteria, such as *Staphylococcus aureus* SA23033004 and *Streptococcus suis* L915 were collected and stored in our laboratory. The colony counting method was used to determine the bacterial number. Bacterial genomic DNA was rapidly extracted from the isolates via MightyPrep reagent (TaKaRa Bio, Kusatsu, Shiga, Japan).

### 2.2 Primer design

The genome sequences of the biomarkers *iroB*, *iucA*, *peg-344* and plasmid-borne *rmpA* carried by the HvKP strain NTUH-K2044 (accession number AP006726) were retrieved from GenBank (National Library of Medicine, Bethesda, MD, USA). On the basis of the DNA sequences of *iroB* with 1,116 bp, *iucA* with 1791 bp, *peg-344* with 903 bp and plasmid-borne *rmpA* with 633 bp, primers were designed via Oligo 7 under default settings (Molecular Biology Insights, Colorado Springs, CO, USA). And primers for these biomarkers in the published literatures were also retrieved. The primer sets were synthesized, purified and confirmed by Sangon Biotech (Shanghai, China).

### 2.3 Establishment of singleplex and duplex PCR

PCR was performed via a CFX96 real-time PCR detection system (Bio-Rad Laboratories, Hercules, CA, USA). Each singleplex and duplex PCR was performed in triplicate, with a final reaction mixture of 20 μL containing 10 μL of 2 × SYBR Green I master mix (Tolo Biotech, Shanghai, China), 2 μL of the DNA template, an appropriate volume of primers and PCR water. The primers were used at a final concentration of 0.2 to 0.5 μM. A two-step amplification protocol was applied, which preceded the initial denaturation at 95°C for 5 min, followed by 35 cycles of 95°C for 15 s and 58°C for 30 s. Then, a melting curve analysis was used with a ramp speed of 0.1°C/s from 65 to 95°C. The LOD in singleplex and duplex PCR was defined as the lowest amount of HvKP that can produce a melting curve with one or two peaks which distinguished from the background noise. The primers for each target in singleplex real-time PCR were screened on the basis of the LOD and specificity. And the primers used in the duplex PCR melting curve assay were screened via two distinguishable melting peaks appeared or not. In addition, DNA templates extracted from other pathogens were used to evaluate the specificity of the developed singleplex and duplex PCRs.

### 2.4 Detection of HvKP in clinical specimens

BALB/c female mice, 6–8 weeks old, 16–18 grams in body weight were obtained from Huaxing Experimental Animal Farm (Zhengzhou, China) and quarantined for 5 days before use. HvKP strains 20 K-368 and 19 K-29; cKP strains 19 K-1018, 19 K-1024 and 19 K-1028; and *E. coli* strain EC23030102, *S. typhimurium* strain SQ1, *S. aureus* strain SA23033004, *S. suis* strain L915 at 10^3^ cfu were used in the intraperitoneal challenge of nine BALB/c mice, respectively. Tail vein blood samples were collected from mice infected with HvKP strains or non-HvKP isolates at the Animal Care Center of Henan Agricultural University, with all operations strictly abiding by the laws and guidelines of China on the care and use of laboratory animals (under experimental license HNND2024030727). A 10 μL sample was mixed with 90 μL of MightyPrep reagent, and incubated at 95°C for 10 min on a block heater. After centrifugation at 12,000 rpm for 2 min, the supernatant was used directly as the DNA template in the developed singleplex and duplex PCRs. Additionally, the string test and sequencing analysis were used as reference methods for these clinical samples.

## 3 Results

### 3.1 Development of singleplex real-time PCR for biomarkers

To reduce the testing time, rapid DNA extraction within 12 min was applied in this study, and the extraction yield was confirmed to be comparable to that of the classical mini spin column-based method according to our previous report (21). After initial screening of primer sets on the basis of the detection limit and specificity, primers whose amplicons were in the range of 99 to 413 bp, as shown in Table 1, were selected for use in subsequent real-time PCR assays.

**Table 1:** Primers used for singleplex and duplex PCRs.

Gene	Orientation	Primer (5′–3′)	Concentration (nM)		Amplicon size	Amplicon Tm	Reference
			Singleplex PCR	Duplex PCR	(bp)	(°C)	
*iroB*	forward	CTA TGG GCT ATT GTA TCC TGT	500	200	195	85.0	This study
	reverse	ATG TGA CTT TCT TTC CGC AGA					
*iucA*	forward-1	GCT GCG CTA CTT CCC TTA TTA CCT G	500	–	200	86.5	This study
	reverse-1	AGT TAC CTT TTA CGT TCC AGT ACG GAT					
	forward-2	GTT CGT GAA ACC AAA GAC	500	300	413	87.5	This study
	reverse-2	AAC GGT TCA TGA GAC TTA					
*peg-344*	forward	GCC AGC GTC TAT TTC AAC TT	500	200	99	76.0	(18)
	reverse	CGG CAA GTC CTG GGT TTA C					
plasmid-borne *rmpA*	forward-1	AGC ACA AAA GAA ACA TAA GAG TAT TGG TTG ACA GC	500	–	128	80.0	This study
	reverse-1	CCC CGA AAC GTC AAG CCA CAT CCA T					
	forward-2	CAA GGA TGT AAA CAT AGT G	500	500	274	78.5	This study
	reverse-2	CCC TTT AGG ATA AAA CCG					

**Figure 1:**
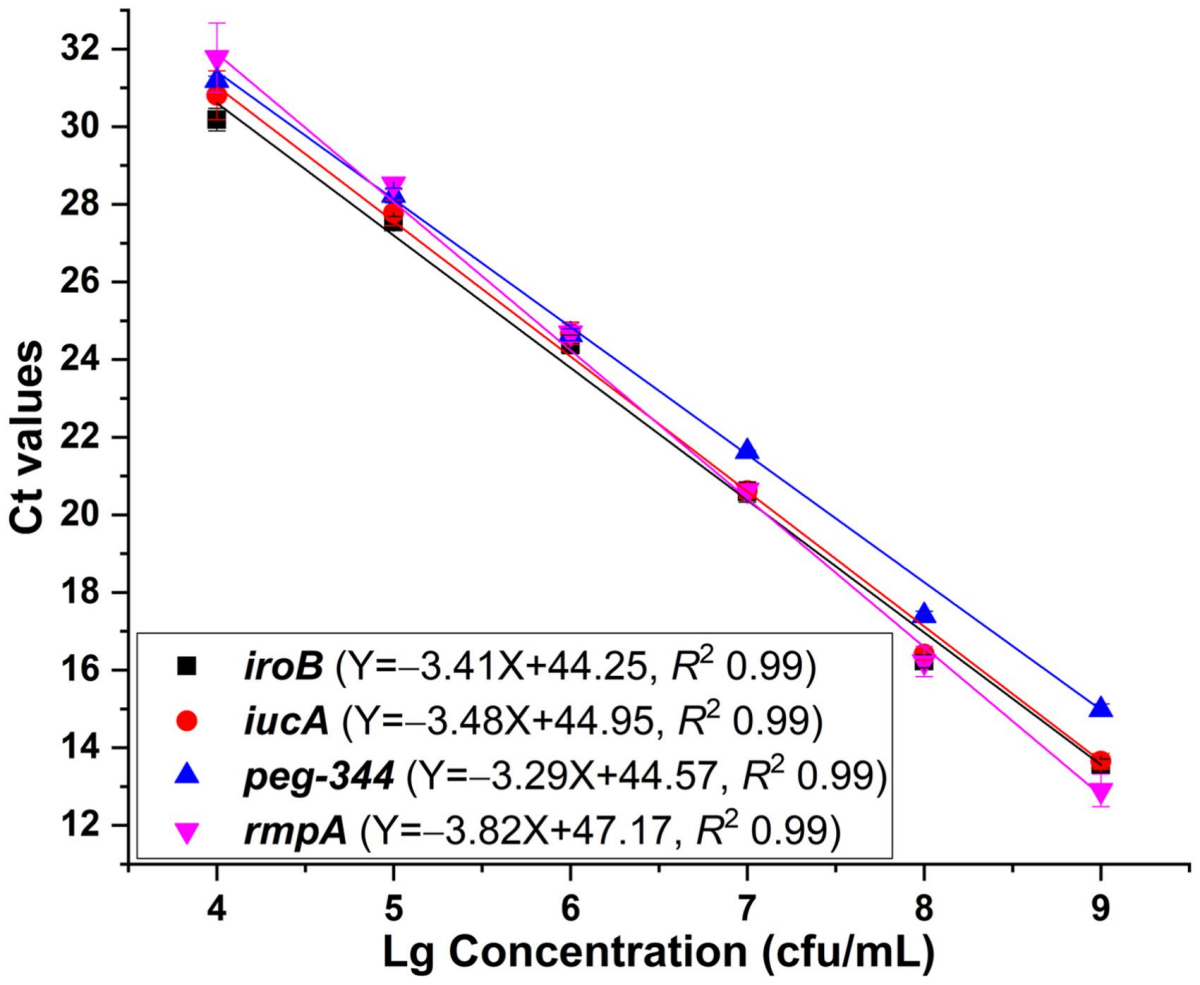
Standard curves generated for *iroB*, *iucA*, *peg-344* and plasmid-borne *rmpA*, respectively via the SYBR Green I-based real-time PCR assays. The primers iucA-1 and rmpA-1 were used for the amplification of *iucA* and plasmid-borne *rmpA*, respectively. PCRs showed efficiencies of 96.5, 93.8, 101.3 and 82.7% for *iroB*, *iucA*, *peg-344* and plasmid-borne *rmpA*, respectively, with all correlation coefficients higher than 0.99.

One HvKP strain termed 20 K-368, which harboring biomarker genes *iroB*, *iucA*, *peg-344* and plasmid-borne *rmpA*, was used in the development of singleplex real-time PCR. The DNA template was extracted from ten-fold serial dilutions of bacterial cultures at concentrations ranging from 1 × 10^2^ to 1 × 10^9^ cfu/mL. The linear range of singleplex real-time PCR was identified as the template concentrations where the corresponding Ct values were fitted into a straight line. As presented in Figure 1, standard curves were fitted with the coefficients of correlation (*R*^2^) greater than 0.99, and the linear ranges were from 10^4^ to 10^9^ cfu/mL for *iroB*, *iucA* (with primer iucA-1 being used), *peg-344* and plasmid-borne *rmpA* (with primer rmpA-1 being used). The efficiency of the developed singleplex real-time PCR method was 96.5% for *iroB*, 93.8% for *iucA*, 101.3% for *peg-344* and 82.7% for plasmid-borne *rmpA*. The LOD was determined to be 10^3^ cfu/mL of HvKP, which was 10 cfu/reaction since 10 μL of bacteria and 90 μL of MightyPrep reagent were mixed for DNA preparation. Melting curve analysis revealed that the average melting temperatures (Tm) of these biomarkers were between 76.0 to 86.5°C (Table 1).

**Figure 2:**
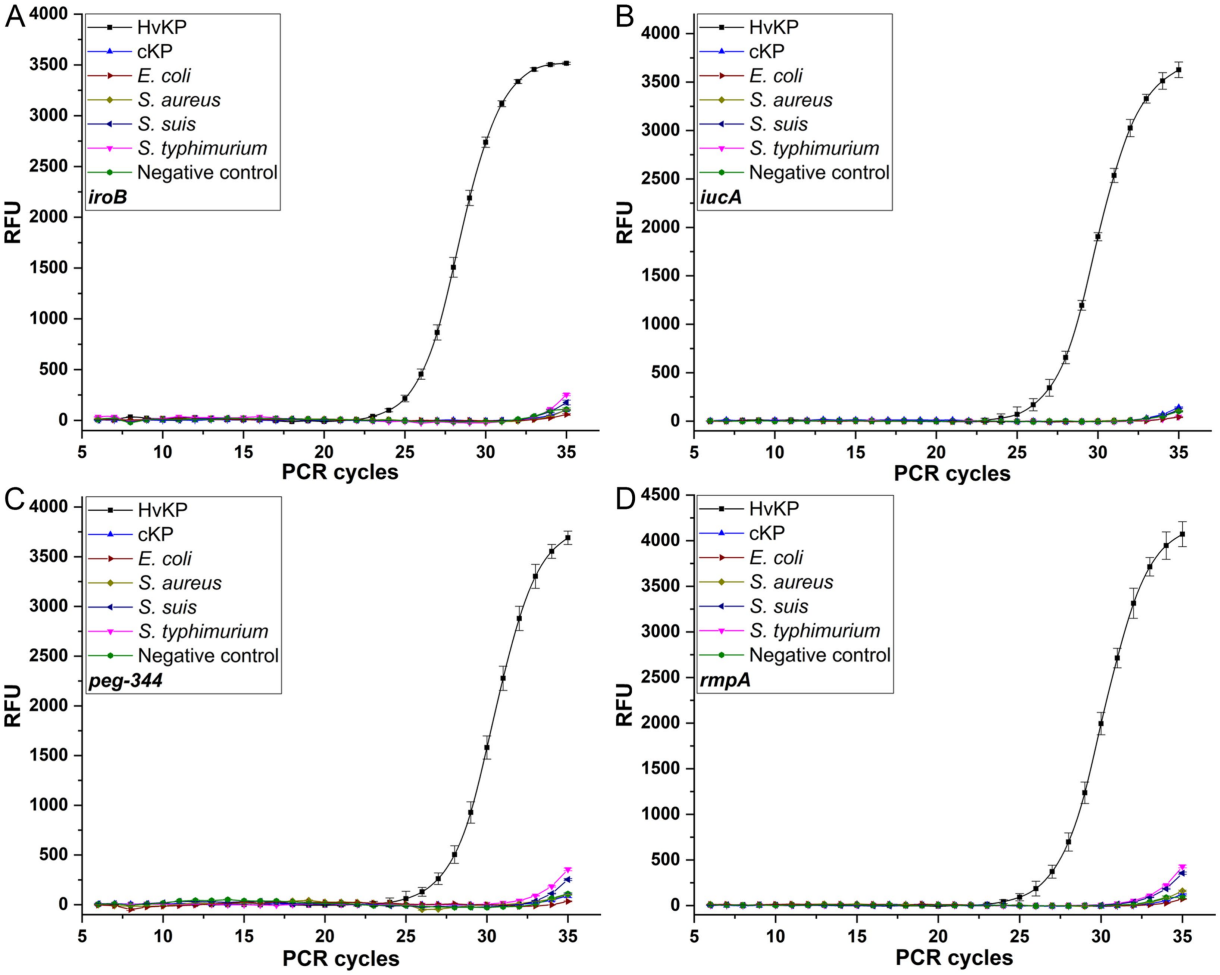
The specificities of the SYBR Green I-based real-time PCR assays for *iroB* **(A)**, *iucA* **(B)**, *peg-344* **(C)** and plasmid-borne *rmpA* **(D)**. An amount of 10^6^ cfu/mL HvKP, cKP, *E. coli*, *S. aureus*, *S. suis* and *S. typhimurium* were used in this study.

The reproducibility was evaluated, with coefficients of variation of no more than 1.26% for *iroB*, no more than 2.04% for *iucA*, no more than 1.00% for *peg-344* and no more than 3.26% for plasmid-borne *rmpA*, which revealed the good repeatability and reproducibility of the established singleplex real-time PCR assays. Moreover, the specificity of the developed singleplex real-time PCR method was measured by testing 10^6^ cfu/mL 19 K-1018 (cKP), EC23030102 (*E. coli*), SA23033004 (*S. aureus*), L915 (*S. suis*) and SQ1 (*S. typhimurium*) isolates. As shown in Figure 2, compared with the signal produced by the 20 K-368 (HvKP) strain, no obvious fluorescent signals were recorded for non-HvKP strains during the amplification process, which highlights the high specificity of these assays for the detection of *iroB*, *iucA*, *peg-344* and plasmid-borne *rmpA* carried by HvKP.

### 3.2 Development of duplex PCR melting curve assays for biomarkers

**Figure 3:**
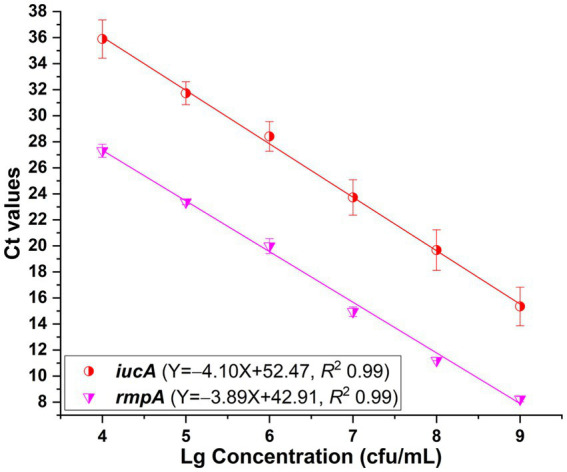
Standard curves plotted for *iucA* and plasmid-borne *rmpA*, respectively in the SYBR Green I-based real-time PCR assays. The primers iucA-2 and rmpA-2 were used for the amplification of *iucA* and plasmid-borne *rmpA*, respectively. PCRs showed efficiencies of 75.3 and 80.7% for *iucA* and plasmid-borne *rmpA*, respectively, with all correlation coefficients higher than 0.99.

In addition to the primers iucA-1 and rmpA-1, singleplex real-time PCR assays were developed with primers iucA-2 and rmpA-2, respectively. Standard curves were fitted with *R*^2^ values greater than 0.99 when 10^4^ to 10^9^ cfu/mL HvKP strain 20 K-368 was used (Figure 3). Analysis shown that the PCR efficiencies were 75.3% for *iucA* and 80.7% for plasmid-borne *rmpA*, which were lower than that of singleplex real-time PCR when the primers iucA-1 and rmpA-1 were used, respectively.

As shown in Table 1, the above developed singleplex real-time PCR and melting curve assays revealed two groups of melting peaks with one at approximately 85.0°C for *iroB* and 86.5–87.5°C for *iucA*, and the other at approximately 76.0°C for *peg-344* and 78.5–80.0°C for plasmid-borne *rmpA*. The main combination criterion of the primers for duplex PCRs was to ensure that two distinguishable melting peaks could be obtained in the mixed reaction. Therefore, one primer of *iroB* or *iucA* in the former group (Tm between 85.0–87.5°C) and the other primer of *peg-344* or plasmid-borne *rmpA* in the latter group (Tm between 76.0–80.0°C) were tried to be mixed in one reaction. After screening, the performance of primer iucA-2 in duplex PCR melting curve assays showed lower detection limits than primer iucA-1 did, as did primer rmpA-2 rather than rmpA-1. In addition, when the primer iroB was mixed with rmpA-1 (or rmpA-2) in one PCR reaction, a melting curve was recorded with only one melting peak, not two different peaks, even when different primer concentrations were attempted, indicating the failure of developing duplex PCR for *iroB* and plasmid-borne *rmpA*. Therefore, primers iroB in combination with peg-344, primers iucA-2 in combination with peg-344, and primers iucA-2 in combination with rmpA-2 were selected for the development of duplex PCRs.

**Figure 4:**
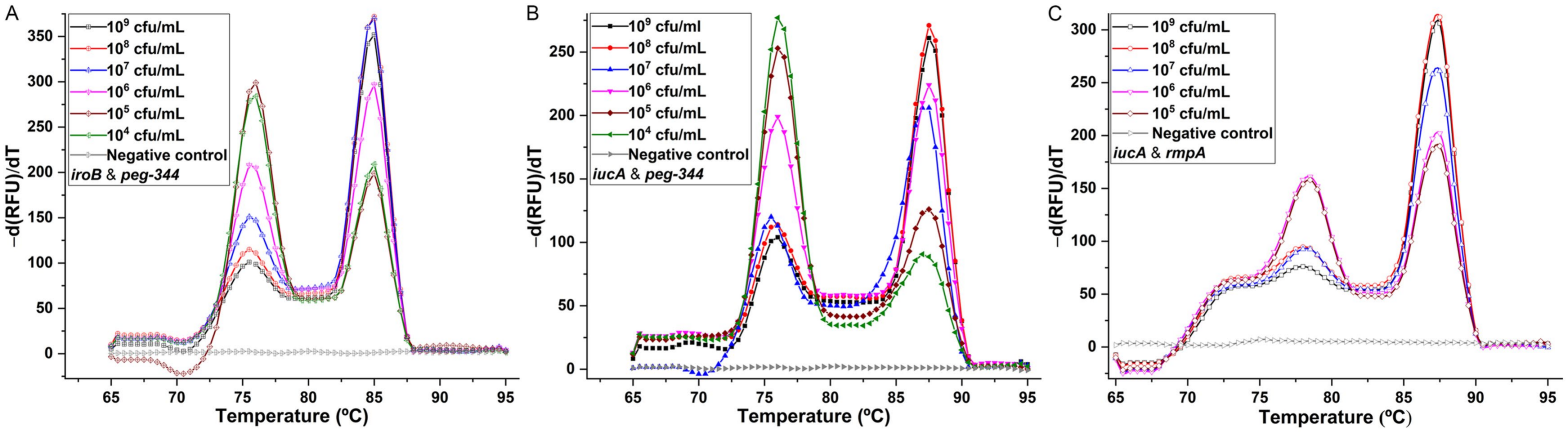
Melting curve analyses for duplex detection of *iroB*/*peg-344* combination **(A)**, *iucA*/*peg-344* combination **(B)**, and *iucA*/*rmpA* combination **(C)**. The primers iroB, iucA-2, peg-344 and rmpA-2 were used for SYBR Green I-based amplification and melting analysis of *iroB*, *iucA*, *peg-344* and plasmid-borne *rmpA*, respectively. The distinguishable and sharp peaks obtained for *iroB* (Tm 85.0°C), *iucA* (Tm 87.5°C), *peg-344* (Tm 76.0°C) and plasmid-borne *rmpA* (Tm 78.5°C) were shown in the duplex PCRs.

To establish the duplex PCR melting curve analysis, the conditions, mainly the primer concentrations were optimized. For singleplex real-time PCR, the concentration was 500 nM for primers iroB, iucA-1, peg-344 and rmpA-1. In duplex PCR for the *iroB*/*peg-344* combination, the concentrations were optimized at 200 nM for primers iroB and peg-344. For the *iucA*/*peg-344* combination, the concentrations were optimized at 300 nM for primer iucA-2 and 200 nM for primer peg-344. And for the *iucA*/*rmpA* combination, the optimal primer concentrations were 300 nM for iucA-2 and 500 nM for rmpA-2. However, the mixing of primers iroB and rmpA-2 in duplex PCR was failed to generate a melting curve with two different peaks. The melting curves for the above three duplex PCR assays were shown in Figure 4. Two distinct melting peaks can be seen in a dilution range of 10^4^ to 10^9^ cfu/mL for the *iroB* (Tm 85.0°C)/*peg-344* (Tm 76.0°C) combination and the *iucA* (Tm 87.5°C)/*peg-344* (Tm 76.0°C) combination, and 10^5^ to 10^9^ cfu/mL for the *iucA* (Tm 87.5°C)/*rmpA* (Tm 78.5°C) combination. That is, for an unknown sample, if melting peaks appear at 85.0 or 76.0°C in duplex PCR for the *iroB*/*peg-344* combination, it indicates that this sample contains the virulence-related genes *iroB* or *peg-344.* The LODs of the duplex PCR melting curve assays for *iroB*/*peg-344*, *iucA*/*peg-344* and *iucA*/*rmpA* combination were 10^4^, 10^4^ and 10^5^ cfu/mL, respectively, which were 10^2^, 10^2^ and 10^3^ cfu/reaction.

**Figure 5:**
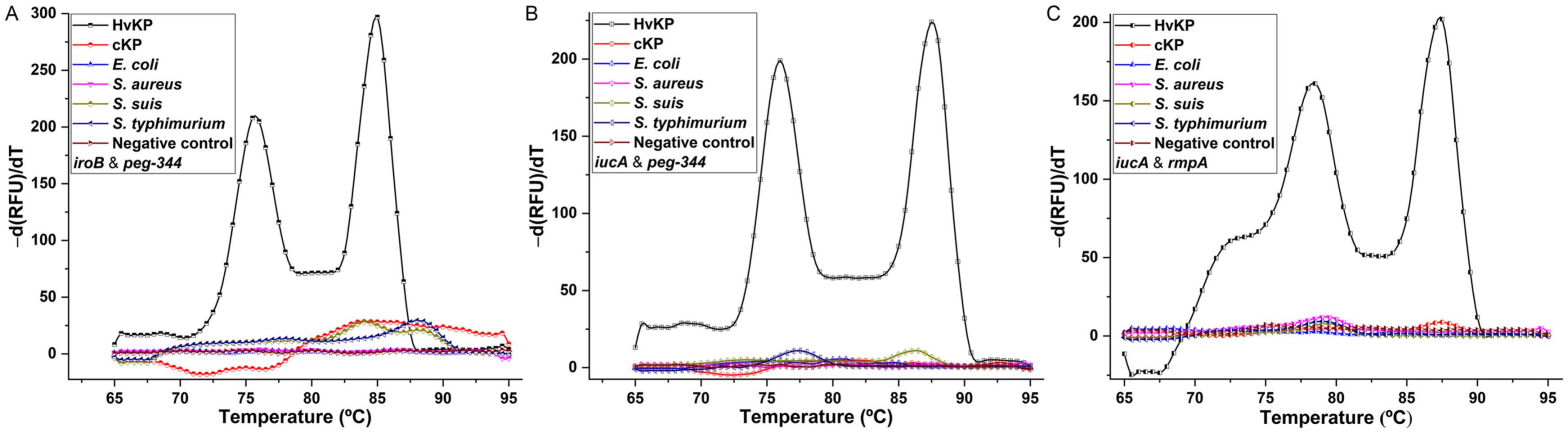
The specificities of melting curve analyses for duplex detection of *iroB*/*peg-344* combination **(A)**, *iucA*/*peg-344* combination **(B)**, and *iucA*/*rmpA* combination **(C)**. An amount of 10^6^ cfu/mL HvKP, cKP, *E. coli*, *S. aureus*, *S. suis* and *S. typhimurium* were used in this study.

The specificities of the developed duplex PCRs were determined by testing for HvKP-positive, and other Gram-positive and Gram-negative pathogens, and the results were shown in Figure 5. Compared with two clearly distinguishable melting peaks generated from the 10^6^ cfu/mL 20 K-368 (HvKP) strain, no obvious melting peaks were recorded from non-HvKP strains, including 10^6^ cfu/mL 19 K-1018 (cKP), EC23030102 (*E. coli*), SA23033004 (*S. aureus*), L915 (*S. suis*) and SQ1 (*S. typhimurium*) isolates. These results suggest that the developed duplex PCR melting curve analyses are highly specific for the detection and differentiation of *iroB*, *iucA*, *peg-344* and plasmid-borne *rmpA* carried by HvKP.

### 3.3 Evaluation of singleplex and duplex PCR in clinical samples

The developed singleplex and duplex PCR assays were used to analyze blood samples from nine infected mice. The results from the developed singleplex and duplex PCR assays revealed that seven out of nine mice were negative for the biomarkers *iroB*, *iucA*, *peg-344* and plasmid-borne *rmpA*, which were recorded as non-HvKP infection. The other two samples were recorded as HvKP infection. The results of singleplex and duplex PCRs showed that four melting peaks corresponding to genes *iroB*, *iucA*, *peg-344*, plasmid-borne *rmpA* were produced in the detection of strain 20 K-368 infection; and two melting peaks corresponding to genes *iucA*, plasmid-borne *rmpA* were generated in the detection of strain 19 K-29 infection, which depicting the high specificity of developed PCR methods. The typical clinical symptoms observed in these two mice were poor body condition, a ruffled hair coat, ocular swelling and redness. The above PCR results were confirmed by isolation of bacterial pathogens from blood samples, and identification through sequencing and the string test. These detection results supported the reliability of the established singleplex and duplex PCRs for the rapid detection of HvKP.

## 4 Discussion

Since the emergence of HvKP in the mid-1980s in Asia, serious and life-threatening infections caused by HvKP have been reported in countries and regions around the world in recent years (22–24). As a notorious zoonotic pathogen, HvKP can be isolated not only from humans but also from livestock, pets, wildlife and the environment (9, 25–28). Notably, the convergent *K. pneumoniae* clones with multidrug-resistant, hypervirulent and highly transmissible profiles are emerging and are now posing unprecedented threats and challenges to public health (29–31). Therefore, methods to identify and surveil the spread of HvKP are urgently needed.

A culture-based string test of *K. pneumoniae* on a blood-agar plate has been widely used to define HvKP when the viscous string is longer than 5 mm from the surface of the plate (1, 23). However, the correlation between the string test and clinically defined HvKP ranged from 51 to 98%, suggesting that more accurate methods are needed for the identification of HvKP (1). Other methods, such as *Galleria mellonella* or mouse lethality tests, qualitative plate siderophore production assay, and serum killing assay have been developed for identifying HvKP (11). However, these methods are labor-intensive, time-consuming, costly and are not suitable for the rapid and simple identification of HvKP.

Molecular-based PCR with the advantages of being timely and cost effective has attracted increasing attention (32–37). However, the challenge is which biomarkers can be used to define HvKP. One study revealed that *iroB*, *iucA*, *peg-344*, plasmid-borne *rmpA* and plasmid-borne *rmpA2* are biomarkers for defining HvKP with accuracies ranging from 95 to 97% (12). Further studies revealed that the presence of four or more of the above-mentioned biomarkers can be used to predict one isolate as HvKP with an accuracy of 84%, and the presence of all five biomarkers was more accurate for the definition of HvKP, with an accuracy of 94% (19, 20). Among these five biomarkers, plasmid-borne *rmpA* (encoding RmpA) and plasmid-borne *rmpA2* (encoding RmpA2), with identities of 84.9% for nucleotides (covering 97%) and 81.3% for proteins (covering 46%), regulate capsular polysaccharide biosynthesis in HvKP. When a total of 1767 sequenced *K. pneumoniae* isolates were analyzed in our laboratory, 26.0% (459/1767) of the isolates carried plasmid-borne *rmpA*. Further analysis revealed that 98.3% (451/459) of the isolates coharbored both plasmid-borne *rmpA* and plasmid-borne *rmpA2*, which suggests that almost all plasmid-borne *rmpA*-positive isolates were positive for plasmid-borne *rmpA2*. To reduce the detection cost, plasmid-borne *rmpA* (other than both plasmid-borne *rmpA* and plasmid-borne *rmpA2*) and other biomarkers *iroB*, *iucA* and *peg-344* were used as the PCR targets for the identification of HvKP in this study.

Recently, singleplex real-time PCR methods for the detection of *iucA*, *peg-344* and *rmpA*, respectively have been reported, but their LODs and amplification efficiencies have not been reported (16). Since the primers used for *rmpA* target the consensus sequences of plasmid-borne *rmpA*, plasmid-borne *rmpA2* and chromosome-borne *rmpA*, the developed singleplex PCR method can detect the above-mentioned three *rmpA* genes simultaneously. The other one singleplex real-time PCR method was developed for *peg-344* with an amplification efficiency of 97.1% (18). The same primer was used in this study for the detection of *peg-344*, and an efficiency of 101.3% was obtained, confirming the robustness of the reported singleplex real-time PCR assay for *peg-344*. To reduce the cost and detection time, duplex PCR methods with the capacity to detect two targets simultaneously were developed in this study. Like in previous studies, melting curve analyses were used for the detection of *iroB*/*peg-344*, *iucA*/*peg-344* and *iucA*/*rmpA* combinations, respectively (38, 39). The LOD of the duplex PCR melting curve assay for *iucA*/*rmpA* was not as low as those of the other two combinations. Further studies are needed to improve the LOD. In the study for triplex detection of *Bacillus cereus*, *Listeria monocytogenes* and *S. aureus* through melting curve analysis, the SYTO9 dye was applied, and an LOD of 3.7 × 10^2^ cfu/mL was achieved, which implies that superior fluorescent DNA intercalating dyes, such as EvaGreen and SYTO9, with less PCR inhibition other than SYBR Green I can be applied in subsequent studies (39). Recently, a quadruplex real-time PCR was developed for the detection of *iroB*, *iucA*, plasmid-borne *rmpA* and plasmid-borne *rmpA2* with molecular beacon probes labeled with the dyes ROX, VIC, FAM and Cy5, respectively (40). An LOD of 1.5 × 10^3^ cfu/mL was obtained, which is lower than that reported in this study. In the other one quadruplex real-time PCR, in which the targets include *iucA* and plasmid-borne *rmpA*/*rmpA2* with molecular beacon probes labeled with the dyes ROX and VIC, respectively, an LOD of 20 cfu/reaction was acquired (41). Through these studies, molecular beacon probes based real-time PCR other than melting curve analysis can be employed to improve the detection limit, but the costs increase. Other methods, such as CRISPR/Cas-based diagnostic tools and recombinase-aided amplification assay have also been developed, and their advantages of ease of use, portability and rapidity are now attracting increasing attention (21, 42–49).

In summary, as shown in Table 2, traditional methods, including *in vivo* models and phenotypic tests, are commonly used for differentiating HvKP strains from cKP. Among these methods, the murine infection model is recognized as the standard. The median lethal dose (LD_50_) of HvKP is generally between 10–10^6^ cfu after intraperitoneal or subcutaneous challenge, whereas the LD_50_ of cKP is usually greater than 10^7^ cfu. Other methods, such as quantitative mucoviscosity assay, serum killing assay, siderophore production assessment and string test, are based on the hypermucoviscous phenotype of HvKP. However, not all HvKP strains are hypermucoviscous, which limits the accuracy and specificity (60.0–94.0%) of these methods. Additionally, complex operation, high cost and time consuming are the main disadvantages that limit the usage of these traditional tests. PCR-based tests, especially multiplex PCR, which target the biomarkers *iroB*, *iucA*, *peg-344*, plasmid-borne *rmpA* and *rmpA2* of HvKP, are easy to use, accurate, specific and time-saving. The developed PCR methods can detect as few as 2 cfu of HvKP, and the specificity can reach 100%. In addition, CRISPR/Cas-based detection, loop-mediated isothermal amplification (LAMP) and recombinase-aided amplification (RAA) provide alternative methods for rapid and specific detection of HvKP.

**Table 2:** Comparison of developed real-time PCRs with several published methods for the identification of HvKP.

Methods	Targets	Detection indicators	Assay time	Costs	LOD	Specificity	References
*In vivo* models							
*Galleria mellonella* infection model	Larvae	≥ 50% Mortality within 7 days after infected with 10^6^ cfu in 10 μL isolate	~7 d	Purchase *G. mellonella*, biosafety cabinet, incubator, etc.	– ^a^	31.0%	(11, 50)
Murine infection model	CD1 mouse	Median lethal dose between 10–10^6^ cfu after intraperitoneal or subcutaneous challenged with isolate	~14 d	Purchase CD1 mouse, animal facility, biosafety cabinet, incubator, etc.	– ^a^	Standard method	(20, 51)
Phenotypic tests							
Quantitative mucoviscosity assay	Isolate	> 0.15 of Post/prespin OD_600_ ratio of isolate culture	~24 h	Purchase biosafety cabinet, centrifuge, incubator, spectrophotometer etc.	– ^a^	93.3%/86.7% (grown in LB or c-M9-te broth)	(51)
Serum killing assay	Isolate	Serum-resistant grade 5 or 6	~16 h	Purchase biosafety cabinet, incubator, spectrophotometer etc.	– ^a^	60.0%	(52, 53)
Siderophore production assessment	Isolate	≥ 30 μg/mL of Total siderophore production from isolate	~16 h	Purchase required reagents, biosafety cabinet, centrifuge, incubator, etc.	– ^a^	94.0%	(12, 51)
String test	Isolate	Viscous string > 5 mm in length from a bacterial colony	~16 h	Purchase blood agar plates, inoculating loops, biosafety cabinet, incubator, etc.	– ^a^	91.0%	(12, 50)
PCR-based tests							
Singleplex PCR	Gene *iroB*	Specific band on gel	~4 h	Purchase block heater, centrifuge, DNA electrophoresis cell, gel imaging system, thermal cycler, etc.	– ^a^	98.1%	(40)
	Gene *iucA*					92.7%	
	Gene plasmid-borne *rmpA*					100%	
	Gene plasmid-borne *rmpA2*					95.6%	
Singleplex PCR	Gene *peg-344*	Specific band on gel	~4 h	Purchase DNA electrophoresis cell, gel imaging system, thermal cycler, etc.	2 × 10^3^ copies plasmid	100%	(49)
	Gene *rmpA*						
Duplex PCR	Gene *peg-344*, plasmid-borne *rmpA*	Specific bands on gel	~4 h	Purchase block heater, centrifuge, DNA electrophoresis cell, gel imaging system, thermal cycler, etc.	12.5 ng genomic DNA	– ^a^	(51)
Triplex PCR	Gene *iucA*, plasmid-borne *rmpA2*, *terB*						
Quadruplex PCR	Gene *gapA*, *iroB*, *irp2*, *iucA*						
Quintuplex PCR	IncHIB replicon gene, *iroN*, *iutA*, plasmid-borne *rmpA*, *rmpA2*	Specific bands on gel	~4 h	Purchase DNA electrophoresis cell, gel imaging system, thermal cycler, water bath, etc.	– ^a^	High specificity	(14)
Nonuplex PCR	Gene *allS*, *entB*, *iutA*, *kfu*, *magA*, *mrkD*, plasmid-borne *rmpA*, *wzi*, *ybtS*	Specific bands on gel	~4 h	Purchase centrifuge, DNA electrophoresis cell, gel imaging system, thermal cycler, water bath, etc.	2 cfu	High specificity	(15)
Real-time PCR-based tests							
Singleplex real-time PCR	Gene *iroB*	Ct value and melting peak	~2 h	Purchase block heater, centrifuge, thermal cycler, etc.	10 cfu	High specificity	This study
	Gene *iucA*						
	Gene *peg-344*						
	Gene plasmid-borne *rmpA*						
Singleplex real-time PCR	Gene *iucA*	Melting peak	~2 h	Purchase block heater, thermal cycler, etc.	– ^a^	– ^a^	(16)
	Gene *peg-344*						
	Gene *rmpA* (both plasmid-borne *rmpA*, *rmpA2* and chromosome-borne *rmpA*)						
Singleplex real-time PCR	Gene *peg-344*	Ct value and melting peak	~2 h	Purchase thermal cycler, etc.	500 fg genomic DNA	High specificity	(18)
Singleplex real-time PCR	Gene *peg-344*	Ct value	~2 h	Purchase thermal cycler, etc.	200 copies plasmid	– ^a^	(49)
	Gene *rmpA*						
Duplex real-time PCR	Gene *iroB*, *peg-344*	Melting peaks	~2 h	Purchase block heater, centrifuge, thermal cycler, etc.	10^2^ cfu	High specificity	This study
	Gene *iucA*, *peg-344*				10^2^ cfu		
	Gene *iucA*, plasmid-borne *rmpA*				10^3^ cfu		
Quadruplex real-time PCR	Gene *gltA*, *iucA*, *KPC*, *rmpA* (both *rmpA* and *rmpA2*)	Ct values	~2 h	Purchase centrifuge, thermal cycler, etc.	20 cfu	High specificity	(41)
Quadruplex real-time PCR	Gene *iroB*, *iucA*, plasmid-borne *rmpA*, *rmpA2*	Ct values	~2 h	Purchase block heater, centrifuge, thermal cycler, etc.	1.5 × 10^3^ cfu/mL	98.0–98.3%	(40)
Other tests							
Singleplex CRISPR/Cas13a detection	Gene *rmpA*	Control/Test line color	~2 h	Purchase block heater, etc.	10.25 ng/mL genomic DNA	High specificity	(42)
Singleplex LAMP	Gene *iroB*	Color change	~1 h	Purchase block heater, centrifuge, etc.	1.5 × 10^2^ cfu/mL	95.2%	(40)
	Gene *iucA*				1.5 × 10^3^ cfu/mL	95.5%	
Singleplex RAA	Gene *peg-344*	Fluorescent signal	~0.5 h	Purchase thermal cycler, etc.	20 copies plasmid	100%	(49)
	Gene *rmpA*						
Sextuplex CRISPR/Cas12a detection	Gene *iucA*, *iroB*, *peg-344*, plasmid-borne *rmpA*, *rmpA2*, *tet*(A)	Fluorescent signal	~1 h	Purchase block heater, etc.	1 cfu	High specificity	(21)

^a^Not mentioned.

^b^A blank entry means that the item value is the same as that in the above row.

In this report, the PCR targets were the HvKP-specific genes *iroB*, *iucA*, *peg-344* and plasmid-borne *rmpA*. However, when the DNA sequence of *iroB* in HvKP (NTUH-K2044 isolate) was used as the query sequence for NCBI BLAST, *Enterobacter hormaechei* and *E. coli*, which carry this *iroB* gene with an identity greater than 88.1% (query coverage 100%), were identified. Similarly, the *iucA* gene in HvKP (NTUH-K2044 isolate) was found in *E. coli*, with an identity higher than 88.3% (query coverage higher than 94%). These data suggest that for *K. pneumoniae* strains, the developed singleplex and duplex PCR can be used to differentiate HvKP from cKP. However, for an unknown isolate, bacterial identification of which one belongs to *K. pneumoniae* or not is essential. Moreover, more hypervirulent strains of other bacterial species require to be collected and used for further specification test of the developed PCRs.

In conclusion, SYBR Green I-based singleplex real-time PCRs for *iroB*, *iucA*, *peg-344* and plasmid-borne *rmpA*, respectively were developed with the LOD of 10 cfu/reaction. And duplex PCR melting curve analyses for *iroB*/*peg-344*, *iucA*/*peg-344* and *iucA*/*rmpA* combinations were performed with LODs of 10^2^, 10^2^ and 10^3^ cfu/reaction, respectively. These methods were found to be reliable and specific for the identification of HvKP. The highlights are (1) rapid SYBR Green I-based singleplex real-time PCRs for HvKP were developed; (2) new duplex PCR melting curve assays for HvKP were established; (3) the detection limit of the singleplex PCRs was 10 cfu; (4) singleplex and duplex tests provide fast, accurate and specific diagnoses of HvKP infection.

Through the initial evaluation of the developed singleplex and duplex PCRs against HvKP, cKP, *E. coli*, *S. aureus*, *S. suis* and *S. typhimurium*, a specific response was obtained for HvKP, which highlights the high specificity of these PCR methods. Further studies will focus on the collection of more HvKP, cKP and other hypervirulent bacterial strains to evaluate the accuracy, sensitivity and specificity of developed PCRs and the development of molecular beacon-based multiplex real-time PCRs for HvKP and other pathogens.

## Data Availability

The original contributions presented in the study are included in the article/supplementary material, further inquiries can be directed to the corresponding author.

## References

[1] Choby JE Howard-Anderson J Weiss DS. Hypervirulent *Klebsiella pneumoniae* – clinical and molecular perspectives. J Intern Med. (2020) 287:283–300. doi: 10.1111/joim.13007, PMID: 31677303 PMC7057273

[2] Russo TA Marr CM. Hypervirulent *Klebsiella pneumoniae*. Clin Microbiol Rev. (2019) 32:e00001-19. doi: 10.1128/CMR.00001-19, PMID: 31092506 PMC6589860

[3] Wall K Macori G Koolman L Li F Fanning S. *Klebsiella*, a hitherto underappreciated zoonotic pathogen of importance to one health: a short review. Zoonoses. (2023) 3:38. doi: 10.15212/ZOONOSES-2023-0016

[4] Wyres KL Holt KE. *Klebsiella pneumoniae* as a key trafficker of drug resistance genes from environmental to clinically important bacteria. Curr Opin Microbiol. (2018) 45:131–9. doi: 10.1016/j.mib.2018.04.004, PMID: 29723841

[5] Pu D Zhao J Chang K Zhuo X Cao B. "superbugs" with hypervirulence and carbapenem resistance in *Klebsiella pneumoniae*: the rise of such emerging nosocomial pathogens in China. Sci Bull. (2023) 68:2658–70. doi: 10.1016/j.scib.2023.09.040 37821268

[6] Liu X Wu Y Zhu Y Jia P Li X Jia X. Emergence of colistin-resistant hypervirulent *Klebsiella pneumoniae* (CoR-HvKp) in China. Emerg Microbes Infect. (2022) 11:648–61. doi: 10.1080/22221751.2022.2036078, PMID: 35086435 PMC8896207

[7] Yao H Qin S Chen S Shen J Du X-D. Emergence of carbapenem-resistant hypervirulent *Klebsiella pneumoniae*. Lancet Infect Dis. (2018) 18:25. doi: 10.1016/S1473-3099(17)30628-X, PMID: 29102518

[8] Li Y Wang Z Dong H Wang M Qin S Chen S. Emergence of *tet*(X4)-positive hypervirulent *Klebsiella pneumoniae* of food origin in China. LWT. (2023) 173:114280. doi: 10.1016/j.lwt.2022.114280

[9] Mario E Hamza D Abdel-Moein K. Hypervirulent *Klebsiella pneumoniae* among diarrheic farm animals: a serious public health concern. Comp Immunol Microbiol Infect Dis. (2023) 102:102077. doi: 10.1016/j.cimid.2023.102077, PMID: 37844369

[10] Liao W Long D Huang Q Wei D Liu X Wan L. Rapid detection to differentiate hypervirulent *Klebsiella pneumoniae* (hvKp) from classical *K. pneumoniae* by identifying *peg-344* with loop-mediated isothermal amplication (LAMP). Front Microbiol. (2020) 11:1189. doi: 10.3389/fmicb.2020.01189, PMID: 32655515 PMC7325879

[11] Zhang Q-B Zhu P Zhang S Rong Y-J Huang Z-A Sun L-W. Hypervirulent *Klebsiella pneumoniae* detection methods: a minireview. Arch Microbiol. (2023) 205:326. doi: 10.1007/s00203-023-03665-y, PMID: 37672079

[12] Russo TA Olson R Fang C-T Stoesser N Miller M MacDonald U. Identification of biomarkers for differentiation of hypervirulent *Klebsiella pneumoniae* from classical *K. pneumoniae*. J Clin Microbiol. (2018) 56:e00776-18. doi: 10.1128/JCM.00776-18, PMID: 29925642 PMC6113484

[13] Yang S Rothman RE. PCR-based diagnostics for infectious diseases: uses, limitations, and future applications in acute-care settings. Lancet Infect Dis. (2004) 4:337–48. doi: 10.1016/S1473-3099(04)01044-8, PMID: 15172342 PMC7106425

[14] Yu F Lv J Niu S Du H Tang Y-W Pitout JDD. Multiplex PCR analysis for rapid detection of *Klebsiella pneumoniae* carbapenem resistant (sequence type 258 [ST258] and ST11) and hypervirulent (ST23, ST65, ST86, and ST375) strains. J Clin Microbiol. (2018) 56:e00731-18. doi: 10.1128/JCM.00731-18, PMID: 29925644 PMC6113471

[15] Compain F Babosan A Brisse S Genel N Audo J Ailloud F. Multiplex PCR for detection of seven virulence factors and K1/K2 capsular serotypes of *Klebsiella pneumoniae*. J Clin Microbiol. (2014) 52:4377–80. doi: 10.1128/JCM.02316-14, PMID: 25275000 PMC4313302

[16] Parrott AM Shi J Aaron J Green DA Whittier S Wu F. Detection of multiple hypervirulent *Klebsiella pneumoniae* strains in a new York City hospital through screening of virulence genes. Clin Microbiol Infect. (2021) 27:583–9. doi: 10.1016/j.cmi.2020.05.012, PMID: 32461145

[17] Turton JF Perry C Elgohari S Hampton CV. PCR characterization and typing of *Klebsiella pneumoniae* using capsular type-specific, variable number tandem repeat and virulence gene targets. J Med Microbiol. (2010) 59:541–7. doi: 10.1099/jmm.0.015198-0, PMID: 20110386

[18] Bulger J MacDonald U Olson R Beanan J Russo TA. Metabolite transporter PEG344 is required for full virulence of hypervirulent *Klebsiella pneumoniae* strain hvKP1 after pulmonary but not subcutaneous challenge. Infect Immun. (2017) 85:e00093-17. doi: 10.1128/IAI.00093-17, PMID: 28717029 PMC5607406

[19] Russo TA Alvarado CL Davies CJ Drayer ZJ Carlino-MacDonald U Hutson A. Differentiation of hypervirulent and classical *Klebsiella pneumoniae* with acquired drug resistance. MBio. (2024) 15:e02867-23. doi: 10.1128/mbio.02867-23, PMID: 38231533 PMC10865842

[20] Russo TA Lebreton F McGann PT. A step forward in hypervirulent *Klebsiella pneumoniae* diagnostics. Emerg Infect Dis. (2025) 31:1–3. doi: 10.3201/eid3101.241516, PMID: 39714290 PMC11682795

[21] Li C Wu Y Chen Y Xu C Yao H Yu W. Violet phosphorene nanosheets coupled with CRISPR/Cas12a in a biosensor with a low background signal for onsite detection of tigecycline-resistant hypervirulent *Klebsiella pneumoniae*. Sens Actuators B Chem. (2023) 395:134509. doi: 10.1016/j.snb.2023.134509

[22] Arcari G Carattoli A. Global spread and evolutionary convergence of multidrug-resistant and hypervirulent *Klebsiella pneumoniae* high-risk clones. Pathog Glob Health. (2023) 117:328–41. doi: 10.1080/20477724.2022.2121362, PMID: 36089853 PMC10177687

[23] Dai P Hu D. The making of hypervirulent *Klebsiella pneumoniae*. J Clin Lab Anal. (2022) 36:e24743. doi: 10.1002/jcla.24743, PMID: 36347819 PMC9757020

[24] Shon AS Bajwa RPS Russo TA. Hypervirulent (hypermucoviscous) *Klebsiella pneumoniae*: a new and dangerous breed. Virulence. (2013) 4:107–18. doi: 10.4161/viru.22718, PMID: 23302790 PMC3654609

[25] Anzai EK de Souza Junior JC Peruchi AR Fonseca JM Gumpl EK Pignatari ACC. First case report of non-human primates (*Alouatta clamitans*) with the hypervirulent *Klebsiella pneumoniae* serotype K1 strain ST 23: a possible emerging wildlife pathogen. J Med Primatol. (2017) 46:337–42. doi: 10.1111/jmp.12296 28809435

[26] Ma Q Zhu Z Liu Y Wang J Pan Z Yao H. Keeping alert to the hypervirulent K1, K2, K3, K5, K54 and K57 strains of *Klebsiella pneumoniae* within dairy production process. Microbes Infect. (2023) 25:105106. doi: 10.1016/j.micinf.2023.105106, PMID: 36720402

[27] Zhang Z Lei L Zhang H Dai H Song Y Li L. Molecular investigation of *Klebsiella pneumoniae* from clinical companion animals in Beijing, China, 2017–2019. Pathogens. (2021) 10:271. doi: 10.3390/pathogens10030271, PMID: 33673656 PMC7997213

[28] Zou H Zhou Z Berglund B Zheng B Meng M Zhao L. Persistent transmission of carbapenem-resistant, hypervirulent *Klebsiella pneumoniae* between a hospital and urban aquatic environments. Water Res. (2023) 242:120263. doi: 10.1016/j.watres.2023.120263, PMID: 37390655

[29] Dong N Yang X Chan EW-C Zhang R Chen S. *Klebsiella* species: taxonomy, hypervirulence and multidrug resistance. EBioMedicine. (2022) 79:103998. doi: 10.1016/j.ebiom.2022.103998, PMID: 35405387 PMC9010751

[30] Liu C Dong N Chan EWC Chen S Zhang R. Molecular epidemiology of carbapenem-resistant *Klebsiella pneumoniae* in China, 2016–20. Lancet Infect Dis. (2022) 22:167–8. doi: 10.1016/S1473-3099(22)00009-3, PMID: 35092791

[31] Zhao W Li S Schwarz S Li A Yao H Du X-D. Detection of a NDM-5-producing *Klebsiella pneumoniae* sequence type 340 (CG258) high-risk clone in swine. Vet Microbiol. (2021) 262:109218. doi: 10.1016/j.vetmic.2021.109218, PMID: 34481222

[32] Chen N Ye M Xiao Y Li S Huang Y Li X. Development of universal and quadruplex real-time RT-PCR assays for simultaneous detection and differentiation of porcine reproductive and respiratory syndrome viruses. Transbound Emerg Dis. (2019) 66:2271–8. doi: 10.1111/tbed.13276, PMID: 31233656

[33] Deng L He C Zhou Y Xu L Xiong H. Ground transport stress affects bacteria in the rumen of beef cattle: a real-time PCR analysis. Anim Sci J. (2017) 88:790–7. doi: 10.1111/asj.12615, PMID: 27696632

[34] Guo Z Li K Qiao S Chen X-X Deng R Zhang G. Development and evaluation of duplex TaqMan real-time PCR assay for detection and differentiation of wide-type and MGF505-2R gene-deleted African swine fever viruses. BMC Vet Res. (2020) 16:428. doi: 10.1186/s12917-020-02639-2, PMID: 33167979 PMC7654620

[35] Lu S-J Ma M-Y Yan X-G Zhao F-J Hu W-Y Ding Q-W. Development and application of a low-priced duplex quantitative PCR assay based on SYBR Green I for the simultaneous detection of porcine deltacoronavirus and porcine sapelovirus. Vet Med. (2023) 68:106–15. doi: 10.17221/79/2022-VETMED, PMID: 37981902 PMC10581527

[36] Yan Y Cui Y Zhao S Jing J Shi K Jian F. Development of a duplex PCR assay for detecting *Theileria luwenshuni* and *Anaplasma phagocytophilum* in sheep and goats. Exp Appl Acarol. (2021) 85:319–30. doi: 10.1007/s10493-021-00662-y, PMID: 34591210

[37] Zheng LL Jin XH Wei FS Wang CQ Chen HY Wang YB. Simultaneous detection of porcine pseudorabies virus, porcine parvovirus and porcine circovirus type 2 by multiplex real-time PCR and amplicon melting curve analysis using SYBR green I. Vet Med. (2018) 63:358–66. doi: 10.17221/3/2018-VETMED

[38] Barletta F Mercado EH Lluque A Ruiz J Cleary TG Ochoa TJ. Multiplex real-time PCR for detection of *Campylobacter*, *Salmonella*, and *Shigella*. J Clin Microbiol. (2013) 51:2822–9. doi: 10.1128/JCM.01397-13, PMID: 23761159 PMC3754658

[39] Forghani F Wei S Oh D-H. A rapid multiplex real-time PCR high-resolution melt curve assay for the simultaneous detection of *Bacillus cereus*, *listeria monocytogenes*, and *Staphylococcus aureus* in food. J Food Prot. (2016) 79:810–5. doi: 10.4315/0362-028X.JFP-15-428, PMID: 27296430

[40] Cai Y Wang W Liang H Huang Q Qin J Guo Z. Sensitive and specific LAMP and multiplex qRT-PCR assays for detection of hypervirulent *Klebsiella pneumoniae*. Diagn Microbiol Infect Dis. (2025) 111:116684. doi: 10.1016/j.diagmicrobio.2025.116684, PMID: 39818182

[41] Xu Z Li B Jiang Y Huang J Su L Wu W. Development of a quadruple qRT-PCR assay for simultaneous identification of hypervirulent and carbapenem-resistant *Klebsiella pneumoniae*. Microbiol Spectr. (2024) 12:e00719-23. doi: 10.1128/spectrum.00719-23, PMID: 38059628 PMC10783029

[42] Bhattacharjee G Gohil N Khambhati K Gajjar D Abusharha A Singh V. A paper-based assay for detecting hypervirulent *Klebsiella pneumoniae* using CRISPR-Cas13a system. Microchem J. (2024) 203:110931. doi: 10.1016/j.microc.2024.110931

[43] Chang Y Deng Y Li T Wang J Wang T Tan F. Visual detection of porcine reproductive and respiratory syndrome virus using CRISPR-Cas13a. Transbound Emerg Dis. (2020) 67:564–71. doi: 10.1111/tbed.13368, PMID: 31541593

[44] Wang Z Yang P-P Zhang Y-H Tian K-Y Bian C-Z Zhao J. Development of a reverse transcription recombinase polymerase amplification combined with lateral-flow dipstick assay for avian influenza H9N2 HA gene detection. Transbound Emerg Dis. (2019) 66:546–51. doi: 10.1111/tbed.13063, PMID: 30403438

[45] Wang Y Yu F Zhang K Shi K Chen Y Li J. End-point RPA-CRISPR/Cas12a-based detection of *Enterocytozoon bieneusi* nucleic acid: rapid, sensitive and specific. BMC Vet Res. (2024) 20:540. doi: 10.1186/s12917-024-04391-3, PMID: 39614269 PMC11606026

[46] Wu Y Tian K Zhang Y Guo H Li N Wang Z. Rapid and visual detection of *Lawsonia intracellularis* with an improved recombinase polymerase amplification assay combined with a lateral flow dipstick. BMC Vet Res. (2019) 15:97. doi: 10.1186/s12917-019-1841-9, PMID: 30898117 PMC6429820

[47] Zhang K Sun Z Shi K Yang D Bian Z Li Y. RPA-CRISPR/Cas12a-based detection of *Haemophilus parasuis*. Animals. (2023) 13:3317. doi: 10.3390/ani13213317, PMID: 37958075 PMC10648042

[48] Zhao Y Dai J Zhang Z Chen J Chen Y Hu C. CRISPR-Cas13a-based lateral flow assay for detection of bovine leukemia virus. Animals. (2024) 14:3262. doi: 10.3390/ani14223262, PMID: 39595314 PMC11590953

[49] Yan C Zhou Y Du S Du B Zhao H Feng Y. Recombinase-aided amplification assay for rapid detection of hypervirulent *Klebsiella pneumoniae* (hvKp) and characterization of the hvKp pathotype. Microbiol Spectr. (2023) 11:e03984-22. doi: 10.1128/spectrum.03984-22, PMID: 36912637 PMC10100362

[50] Li G Shi J Zhao Y Xie Y Tang Y Jiang X. Identification of hypervirulent *Klebsiella pneumoniae* isolates using the string test in combination with *galleria mellonella* infectivity. Eur J Clin Microbiol Infect Dis. (2020) 39:1673–9. doi: 10.1007/s10096-020-03890-z, PMID: 32318968

[51] Russo TA MacDonald U Hassan S Camanzo E LeBreton F Corey B. An assessment of siderophore production, mucoviscosity, and mouse infection models for defining the virulence spectrum of hypervirulent *Klebsiella pneumoniae*. mSphere. (2021) 6:e00045-00021. doi: 10.1128/mSphere.00045-21 33762316 PMC8546679

[52] Wang T-C Lin J-C Chang J-C Hiaso Y-W Wang C-H Chiu S-K. Virulence among different types of hypervirulent *Klebsiella pneumoniae* with multi-locus sequence type (MLST)-11, serotype K1 or K2 strains. Gut Pathog. (2021) 13:40. doi: 10.1186/s13099-021-00439-z, PMID: 34154656 PMC8218402

[53] Al Ismail D Campos-Madueno EI Dona V Endimiani A. Hypervirulent *Klebsiella pneumoniae* (hvKp): overview, epidemiology, and laboratory detection. Pathog Immun. (2025) 10:80–119. doi: 10.20411/pai.v10i1.777, PMID: 39911145 PMC11792540

